# Bromidobis(*N*,*N*′-diphenyl­thio­urea-κ*S*)copper(I) monohydrate

**DOI:** 10.1107/S1600536809026038

**Published:** 2009-07-11

**Authors:** Muhammad Mufakkar, M. Nawaz Tahir, Saeed Ahmad, Muhammad Ashraf Shaheen, Abdul Waheed

**Affiliations:** a Department of Chemistry, Government College University, Lahore, Pakistan; b Department of Physics, University of Sargodha, Sargodha, Pakistan; c Department of Chemistry, University of Engineering and Technology, Lahore, Pakistan; d Department of Chemistry, University of Sargodha, Sargodha, Pakistan

## Abstract

In the title compound, [CuBr(C_13_H_12_N_2_S)_2_]·H_2_O, the Cu^I^ atom adopts a slightly distorted trigonal-planar coordination arising from two S atoms of two diphenyl­thio­urea ligands and a bromide ion. There are two intra­molecular N—H⋯Br hydrogen bonds completing twisted six-membered rings with *R*(6) motifs. The dihedral angles between the aromatic rings in the ligands are 62.11 (13) and 85.73 (13)°. In the crystal, components are linked by N—H⋯O, O—H⋯S and O—H⋯π inter­actions. There also exist π–π inter­actions with a distance of 3.876 (2) Å between the centroids of benzene rings of two different ligands. Together, the inter­molecular inter­actions lead to a three-dimensional network.

## Related literature

For related structures, see: Khan *et al.* (2007 ▶); Mufakkar *et al.* (2007 ▶); Zoufalá *et al.* (2007 ▶). For graph-set notation, see: Bernstein *et al.* (1995 ▶).
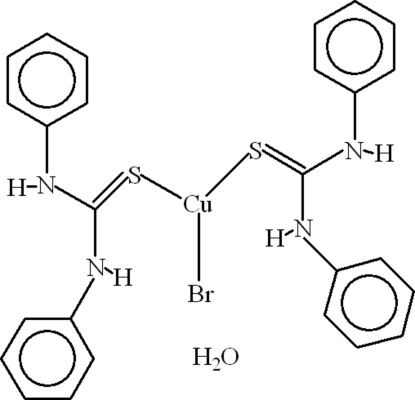


## Experimental

### 

#### Crystal data

[CuBr(C_13_H_12_N_2_S)_2_]·H_2_O
*M* *_r_* = 618.08
Triclinic, 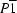

*a* = 9.6195 (5) Å
*b* = 12.1937 (6) Å
*c* = 12.7969 (6) Å
α = 89.345 (2)°
β = 73.154 (1)°
γ = 69.225 (2)°
*V* = 1336.20 (11) Å^3^
*Z* = 2
Mo *K*α radiation
μ = 2.50 mm^−1^
*T* = 296 K
0.28 × 0.23 × 0.20 mm

#### Data collection

Bruker Kappa APEXII CCD diffractometer
Absorption correction: multi-scan (*SADABS*; Bruker, 2005 ▶) *T*_min_ = 0.509, *T*_max_ = 0.606
27804 measured reflections
6568 independent reflections
5426 reflections with *I* > 2σ(*I*)
*R*_int_ = 0.024

#### Refinement

*R*[*F* ^2^ > 2σ(*F* ^2^)] = 0.039
*wR*(*F* ^2^) = 0.122
*S* = 1.04
6568 reflections
316 parameters
H-atom parameters constrained
Δρ_max_ = 0.47 e Å^−3^
Δρ_min_ = −1.27 e Å^−3^

### 

Data collection: *APEX2* (Bruker, 2007 ▶); cell refinement: *SAINT* (Bruker, 2007 ▶); data reduction: *SAINT*; program(s) used to solve structure: *SHELXS97* (Sheldrick, 2008 ▶); program(s) used to refine structure: *SHELXL97* (Sheldrick, 2008 ▶); molecular graphics: *ORTEP-3 for Windows* (Farrugia, 1997 ▶) and *PLATON* (Spek, 2009 ▶); software used to prepare material for publication: *WinGX* (Farrugia, 1999 ▶) and *PLATON*.

## Supplementary Material

Crystal structure: contains datablocks global, I. DOI: 10.1107/S1600536809026038/hb5021sup1.cif

Structure factors: contains datablocks I. DOI: 10.1107/S1600536809026038/hb5021Isup2.hkl

Additional supplementary materials:  crystallographic information; 3D view; checkCIF report

## Figures and Tables

**Table:** 

Cu1—Br1	2.3387 (5)
Cu1—S1	2.2263 (8)
Cu1—S2	2.2129 (8)

**Table:** 

Br1—Cu1—S1	125.03 (3)
Br1—Cu1—S2	126.04 (3)
S1—Cu1—S2	108.93 (3)

**Table 2:** Hydrogen-bond geometry (Å, °)

*D*—H⋯*A*	*D*—H	H⋯*A*	*D*⋯*A*	*D*—H⋯*A*
N1—H1*N*⋯O1^i^	0.86	2.35	3.046 (4)	139
O1—H1*O*⋯S1^ii^	0.83	2.66	3.462 (3)	163
N2—H2*N*⋯Br1	0.86	2.59	3.435 (2)	169
N3—H3*N*⋯O1^iii^	0.86	2.16	2.957 (3)	155
N4—H4*N*⋯Br1	0.86	2.72	3.573 (2)	170
C13—H13⋯N1	0.93	2.58	3.000 (4)	108
C13—H13⋯S2^iv^	0.93	2.86	3.523 (3)	129
O1—H2*O*⋯*CgD*^iii^	0.80	2.78	3.306 (3)	125

Symmetry codes: (i) 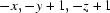; (ii) 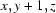; (iii) 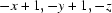; (iv) 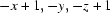. *CgD* is the centroid of the C21–C26 benzene ring.

**Fig. 1.**
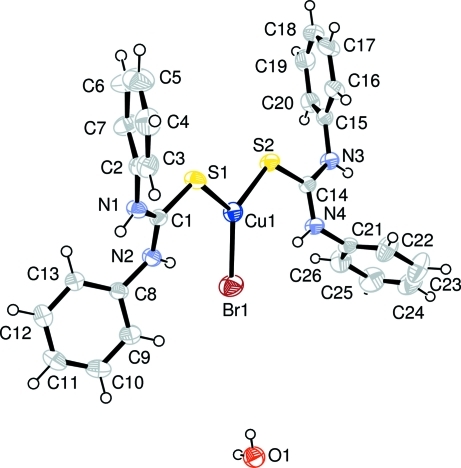
View of (I) with displacement ellipsoids drawn at the 30% probability level. H-atoms are shown by small spheres of arbitrary radius.

**Fig. 2.**
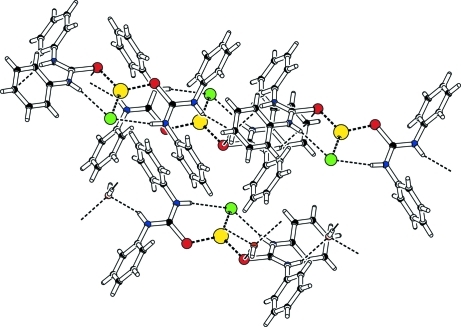
The partial packing of (I), showing that molecules form ring motifs and form three dimensional polymeric network.

## supplementary crystallographic information

### Comment

The crystal structure of tetrakis(N-methylthiourea-S)copper(I) iodide (Mufakkar *et al.*, 2007)), tris(N,N'-dibutylthiourea-S)iodidocopper(I) 0.6-hydrate (Khan *et al.*, 2007) and tetrakis(N-methylthiourea)copper(I) chloride (Zoufalá *et al.*, 2007) have been reported by our group containing substituted thiourea. In continuation to the copper complexes of substituted thiourea, the title compound (I), (Fig. 1) is now reported.

In (I), the copper atom is coordinated to two S-atoms of two diphenylthiourea ligands and the Br-atom.  Cu1 is at a distance of -0.0061 (5) Å from the plane of S1/S2/BR1. In one ligand the benzene rings A (C2—C7) and B (C8—C13) make a dihedral angle of 85.73 (13)°, whereas in the other ligand the benzene rings C (C15—C20) and D (C21—C26) are oriented at dihedral angle of 62.11 (13)°. There exist two intramolecular H-bonds of N–H···Br type, completing two twisted six membered rings with ring motifs R_1_^1^(6) (Bernstein *et al.*, 1995). The NH-groups not involving in H-bonding with Br-atom, make intermolecular H-bonds with O-atom of water. It is interesting that only one H-atom of water molecule make H-bonding with one of S-atom, whereas the other make a π interaction (Table 1). There exist π–π interactions between CgA···CgC^i^ [symmetry code: i = -1 + *x*, *y*, 1 + *z*] and CgC···CgA^ii^ [symmetry code: ii = 1 + *x*, *y*, -1 + *z*] at a distance of 3.876 (2) Å, where CgA and CgC are the centroids of benzene rings A and C, respectively. The molecules are linked each other through H-bonding in the form of three dimensional polymeric network.

### Experimental

Copper(I) bromide (0.14 g, 1.0 mmol) was dissolved in 15 ml of acetonitrile and it was added to two equivalents of N,N'-diphenylthiourea in acetonitrile. White precipitate formed immediately were filtered and the filtrate was kept for crystallization. As a result colourless prisms of (I) were obtained after 24 h.

### Refinement

The H-atoms were positioned geometrically, with N—H = 0.86 Å for NH-groups and C—H = 0.93 Å for aromatic rings. After this the H-atoms of water molecule were taken from difference Fourier map in two steps. All the H-atoms were constrained to ride on their parent atoms, with U_iso_(H) = xU_eq_(C, N, O), where x = 1.2 for all H atoms.

### Crystal data

**Table:**

[CuBr(C_13_H_12_N_2_S)_2_]·H_2_O	*Z* = 2
*M**_r_* = 618.08	*F*(000) = 628
Triclinic, *P*1	*D*_x_ = 1.536 Mg m^−^^3^
Hall symbol: -P 1	Mo *K*α radiation, λ = 0.71073 Å
*a* = 9.6195 (5) Å	Cell parameters from 6568 reflections
*b* = 12.1937 (6) Å	θ = 2.3–28.3°
*c* = 12.7969 (6) Å	µ = 2.50 mm^−^^1^
α = 89.345 (2)°	*T* = 296 K
β = 73.154 (1)°	Prismatic, colourless
γ = 69.225 (2)°	0.28 × 0.23 × 0.20 mm
*V* = 1336.20 (11) Å^3^	

### Data collection

**Table:**

Bruker Kappa APEXII CCD diffractometer	6568 independent reflections
Radiation source: fine-focus sealed tube	5426 reflections with *I* > 2σ(*I*)
graphite	*R*_int_ = 0.024
Detector resolution: 7.40 pixels mm^-1^	θ_max_ = 28.3°, θ_min_ = 2.3°
ω scans	*h* = −12→12
Absorption correction: multi-scan (*SADABS*; Bruker, 2005)	*k* = −16→16
*T*_min_ = 0.509, *T*_max_ = 0.606	*l* = −17→15
27804 measured reflections	

### Refinement

**Table:**

Refinement on *F*^2^	Primary atom site location: structure-invariant direct methods
Least-squares matrix: full	Secondary atom site location: difference Fourier map
*R*[*F*^2^ > 2σ(*F*^2^)] = 0.039	Hydrogen site location: inferred from neighbouring sites
*wR*(*F*^2^) = 0.122	H-atom parameters constrained
*S* = 1.04	*w* = 1/[σ^2^(*F*_o_^2^) + (0.0609*P*)^2^ + 1.6374*P*] where *P* = (*F*_o_^2^ + 2*F*_c_^2^)/3
6568 reflections	(Δ/σ)_max_ < 0.001
316 parameters	Δρ_max_ = 0.47 e Å^−^^3^
0 restraints	Δρ_min_ = −1.27 e Å^−^^3^

### Special details

**Table:**

Geometry. Bond distances, angles etc. have been calculated using the rounded fractional coordinates. All su's are estimated from the variances of the (full) variance-covariance matrix. The cell esds are taken into account in the estimation of distances, angles and torsion angles
Refinement. Refinement of *F*^2^ against ALL reflections. The weighted *R*-factor *wR* and goodness of fit *S* are based on *F*^2^, conventional *R*-factors *R* are based on *F*, with *F* set to zero for negative *F*^2^. The threshold expression of *F*^2^ > σ(*F*^2^) is used only for calculating *R*-factors(gt) *etc*. and is not relevant to the choice of reflections for refinement. *R*-factors based on *F*^2^ are statistically about twice as large as those based on *F*, and *R*- factors based on ALL data will be even larger.

### Fractional atomic coordinates and isotropic or equivalent isotropic displacement parameters (Å^2^)

**Table:**

	*x*	*y*	*z*	*U*_iso_*/*U*_eq_	
Br1	0.33324 (5)	0.31120 (3)	0.30610 (3)	0.0533 (1)	
Cu1	0.44586 (4)	0.10611 (3)	0.26717 (3)	0.0365 (1)	
S1	0.38543 (9)	−0.02233 (6)	0.37916 (6)	0.0371 (2)	
S2	0.62984 (9)	0.00799 (6)	0.11533 (5)	0.0363 (2)	
N1	0.1849 (3)	−0.0049 (2)	0.57745 (18)	0.0329 (6)	
N2	0.2381 (3)	0.1621 (2)	0.52856 (18)	0.0332 (7)	
N3	0.7569 (3)	0.0539 (2)	−0.08459 (18)	0.0333 (6)	
N4	0.5479 (3)	0.2083 (2)	0.02461 (19)	0.0340 (7)	
C1	0.2624 (3)	0.0486 (2)	0.5043 (2)	0.0285 (7)	
C2	0.2046 (3)	−0.1261 (2)	0.5679 (2)	0.0296 (7)	
C3	0.0747 (4)	−0.1555 (3)	0.5850 (3)	0.0389 (9)	
C4	0.0924 (5)	−0.2732 (3)	0.5780 (3)	0.0513 (11)	
C5	0.2378 (5)	−0.3602 (3)	0.5535 (4)	0.0632 (13)	
C6	0.3667 (5)	−0.3298 (3)	0.5391 (4)	0.0621 (13)	
C7	0.3512 (4)	−0.2133 (3)	0.5473 (3)	0.0448 (10)	
C8	0.1611 (3)	0.2336 (2)	0.6302 (2)	0.0312 (8)	
C9	0.0889 (4)	0.3536 (3)	0.6266 (3)	0.0481 (10)	
C10	0.0182 (5)	0.4277 (3)	0.7237 (3)	0.0619 (13)	
C11	0.0184 (5)	0.3834 (3)	0.8227 (3)	0.0564 (11)	
C12	0.0909 (4)	0.2642 (3)	0.8259 (3)	0.0449 (10)	
C13	0.1640 (4)	0.1892 (3)	0.7301 (2)	0.0376 (8)	
C14	0.6462 (3)	0.0959 (2)	0.0114 (2)	0.0284 (7)	
C15	0.8697 (3)	−0.0636 (3)	−0.1082 (2)	0.0321 (7)	
C16	0.8232 (4)	−0.1582 (3)	−0.1121 (3)	0.0410 (9)	
C17	0.9331 (5)	−0.2719 (3)	−0.1320 (3)	0.0536 (11)	
C18	1.0894 (4)	−0.2896 (3)	−0.1513 (3)	0.0552 (11)	
C19	1.1351 (4)	−0.1968 (3)	−0.1499 (3)	0.0533 (10)	
C20	1.0255 (3)	−0.0819 (3)	−0.1277 (3)	0.0413 (9)	
C21	0.5400 (3)	0.2845 (2)	−0.0615 (2)	0.0337 (8)	
C22	0.4526 (4)	0.2811 (3)	−0.1278 (3)	0.0557 (12)	
C23	0.4443 (5)	0.3550 (4)	−0.2108 (4)	0.0703 (17)	
C24	0.5207 (5)	0.4323 (3)	−0.2251 (3)	0.0628 (14)	
C25	0.6103 (5)	0.4334 (4)	−0.1598 (4)	0.0647 (14)	
C26	0.6218 (5)	0.3594 (3)	−0.0775 (3)	0.0514 (11)	
O1	0.1532 (3)	0.8554 (2)	0.29920 (19)	0.0501 (8)	
H1N	0.11673	0.03785	0.63566	0.0395*	
H2N	0.27483	0.19654	0.47431	0.0399*	
H3	−0.02407	−0.09678	0.60112	0.0466*	
H3N	0.76077	0.10051	−0.13542	0.0399*	
H4	0.00491	−0.29344	0.59005	0.0615*	
H4N	0.48508	0.23679	0.08933	0.0408*	
H5	0.24930	−0.43899	0.54660	0.0755*	
H6	0.46526	−0.38872	0.52367	0.0746*	
H7	0.43836	−0.19341	0.53904	0.0537*	
H9	0.08789	0.38413	0.55980	0.0576*	
H10	−0.02992	0.50831	0.72171	0.0742*	
H11	−0.03026	0.43368	0.88744	0.0678*	
H12	0.09072	0.23412	0.89305	0.0539*	
H13	0.21504	0.10909	0.73246	0.0452*	
H16	0.71862	−0.14548	−0.10142	0.0492*	
H17	0.90225	−0.33603	−0.13232	0.0645*	
H18	1.16340	−0.36590	−0.16526	0.0663*	
H19	1.24041	−0.20984	−0.16395	0.0639*	
H20	1.05705	−0.01834	−0.12595	0.0495*	
H22	0.39907	0.22959	−0.11736	0.0667*	
H23	0.38646	0.35207	−0.25697	0.0844*	
H24	0.51177	0.48381	−0.27901	0.0757*	
H25	0.66422	0.48465	−0.17083	0.0781*	
H26	0.68364	0.36004	−0.03368	0.0618*	
H1O	0.22282	0.86957	0.31621	0.0601*	
H2O	0.19151	0.78537	0.29707	0.0601*	

### Atomic displacement parameters (Å^2^)

**Table:**

	*U*^11^	*U*^22^	*U*^33^	*U*^12^	*U*^13^	*U*^23^
Br1	0.0631 (2)	0.0397 (2)	0.0533 (2)	−0.0185 (2)	−0.0129 (2)	0.0145 (2)
Cu1	0.0378 (2)	0.0381 (2)	0.0288 (2)	−0.0137 (2)	−0.0038 (1)	0.0081 (1)
S1	0.0439 (4)	0.0281 (3)	0.0275 (3)	−0.0113 (3)	0.0041 (3)	0.0020 (2)
S2	0.0398 (4)	0.0300 (3)	0.0285 (3)	−0.0075 (3)	−0.0020 (3)	0.0107 (3)
N1	0.0369 (12)	0.0270 (11)	0.0266 (10)	−0.0108 (10)	0.0011 (9)	0.0016 (8)
N2	0.0428 (13)	0.0266 (11)	0.0262 (10)	−0.0131 (10)	−0.0043 (9)	0.0043 (8)
N3	0.0330 (11)	0.0321 (12)	0.0272 (10)	−0.0096 (10)	−0.0013 (9)	0.0078 (9)
N4	0.0356 (12)	0.0284 (11)	0.0280 (11)	−0.0072 (10)	−0.0010 (9)	0.0072 (9)
C1	0.0285 (12)	0.0262 (12)	0.0266 (12)	−0.0073 (10)	−0.0060 (9)	0.0046 (9)
C2	0.0346 (13)	0.0298 (13)	0.0237 (11)	−0.0129 (11)	−0.0065 (10)	0.0062 (9)
C3	0.0377 (15)	0.0432 (16)	0.0379 (15)	−0.0174 (13)	−0.0118 (12)	0.0095 (12)
C4	0.061 (2)	0.055 (2)	0.0535 (19)	−0.0380 (18)	−0.0198 (16)	0.0120 (16)
C5	0.081 (3)	0.0372 (18)	0.070 (2)	−0.0297 (19)	−0.011 (2)	0.0072 (17)
C6	0.052 (2)	0.0324 (17)	0.082 (3)	−0.0068 (15)	−0.0023 (19)	0.0101 (17)
C7	0.0370 (15)	0.0352 (16)	0.0577 (19)	−0.0126 (13)	−0.0093 (14)	0.0148 (14)
C8	0.0323 (13)	0.0262 (13)	0.0332 (13)	−0.0089 (10)	−0.0093 (10)	0.0003 (10)
C9	0.066 (2)	0.0290 (15)	0.0455 (17)	−0.0106 (14)	−0.0203 (16)	0.0045 (12)
C10	0.079 (3)	0.0293 (16)	0.061 (2)	0.0008 (17)	−0.023 (2)	−0.0063 (15)
C11	0.063 (2)	0.0451 (19)	0.0463 (19)	−0.0051 (17)	−0.0126 (16)	−0.0163 (15)
C12	0.0524 (18)	0.0476 (18)	0.0328 (14)	−0.0154 (15)	−0.0138 (13)	0.0001 (13)
C13	0.0428 (16)	0.0317 (14)	0.0340 (14)	−0.0082 (12)	−0.0122 (12)	0.0029 (11)
C14	0.0277 (12)	0.0293 (13)	0.0276 (12)	−0.0115 (10)	−0.0064 (9)	0.0066 (10)
C15	0.0318 (13)	0.0350 (14)	0.0234 (11)	−0.0086 (11)	−0.0040 (10)	0.0025 (10)
C16	0.0389 (15)	0.0413 (16)	0.0409 (16)	−0.0144 (13)	−0.0095 (12)	−0.0046 (12)
C17	0.070 (2)	0.0355 (17)	0.0500 (19)	−0.0156 (16)	−0.0150 (17)	−0.0070 (14)
C18	0.057 (2)	0.0422 (19)	0.0456 (18)	0.0061 (16)	−0.0153 (16)	−0.0076 (14)
C19	0.0344 (16)	0.065 (2)	0.0468 (18)	−0.0018 (15)	−0.0127 (14)	−0.0062 (16)
C20	0.0356 (15)	0.0489 (18)	0.0384 (15)	−0.0164 (13)	−0.0087 (12)	−0.0002 (13)
C21	0.0342 (14)	0.0269 (13)	0.0326 (13)	−0.0080 (11)	−0.0036 (11)	0.0095 (10)
C22	0.060 (2)	0.061 (2)	0.066 (2)	−0.0357 (19)	−0.0326 (19)	0.0340 (19)
C23	0.078 (3)	0.085 (3)	0.071 (3)	−0.041 (3)	−0.044 (2)	0.044 (2)
C24	0.073 (3)	0.051 (2)	0.054 (2)	−0.0160 (19)	−0.0136 (19)	0.0303 (17)
C25	0.087 (3)	0.051 (2)	0.068 (2)	−0.042 (2)	−0.021 (2)	0.0278 (19)
C26	0.067 (2)	0.0484 (19)	0.0520 (19)	−0.0341 (18)	−0.0217 (17)	0.0159 (15)
O1	0.0479 (13)	0.0506 (14)	0.0404 (12)	−0.0115 (11)	−0.0050 (10)	0.0059 (10)

### Geometric parameters (Å, °)

**Table:**

Cu1—Br1	2.3387 (5)	C15—C20	1.380 (5)
Cu1—S1	2.2263 (8)	C16—C17	1.385 (5)
Cu1—S2	2.2129 (8)	C17—C18	1.387 (7)
S1—C1	1.709 (3)	C18—C19	1.354 (5)
S2—C14	1.705 (2)	C19—C20	1.394 (5)
O1—H1O	0.8300	C21—C22	1.367 (5)
O1—H2O	0.8000	C21—C26	1.381 (5)
N1—C1	1.336 (4)	C22—C23	1.387 (6)
N1—C2	1.424 (3)	C23—C24	1.368 (7)
N2—C1	1.343 (3)	C24—C25	1.367 (7)
N2—C8	1.418 (3)	C25—C26	1.382 (6)
N3—C15	1.428 (4)	C3—H3	0.9300
N3—C14	1.332 (3)	C4—H4	0.9300
N4—C14	1.340 (3)	C5—H5	0.9300
N4—C21	1.435 (3)	C6—H6	0.9300
N1—H1N	0.8600	C7—H7	0.9300
N2—H2N	0.8600	C9—H9	0.9300
N3—H3N	0.8600	C10—H10	0.9300
N4—H4N	0.8600	C11—H11	0.9300
C2—C3	1.377 (5)	C12—H12	0.9300
C2—C7	1.384 (5)	C13—H13	0.9300
C3—C4	1.385 (5)	C16—H16	0.9300
C4—C5	1.371 (6)	C17—H17	0.9300
C5—C6	1.379 (7)	C18—H18	0.9300
C6—C7	1.377 (5)	C19—H19	0.9300
C8—C13	1.387 (4)	C20—H20	0.9300
C8—C9	1.386 (4)	C22—H22	0.9300
C9—C10	1.387 (5)	C23—H23	0.9300
C10—C11	1.372 (5)	C24—H24	0.9300
C11—C12	1.377 (5)	C25—H25	0.9300
C12—C13	1.380 (5)	C26—H26	0.9300
C15—C16	1.383 (5)		
			
Br1—Cu1—S1	125.03 (3)	C22—C21—C26	120.7 (3)
Br1—Cu1—S2	126.04 (3)	N4—C21—C26	119.9 (3)
S1—Cu1—S2	108.93 (3)	N4—C21—C22	119.4 (3)
Cu1—S1—C1	110.49 (9)	C21—C22—C23	119.5 (4)
Cu1—S2—C14	111.94 (9)	C22—C23—C24	120.3 (4)
H1O—O1—H2O	96.00	C23—C24—C25	119.7 (4)
C1—N1—C2	126.0 (2)	C24—C25—C26	120.9 (4)
C1—N2—C8	129.7 (2)	C21—C26—C25	118.9 (4)
C14—N3—C15	124.0 (2)	C2—C3—H3	120.00
C14—N4—C21	124.7 (2)	C4—C3—H3	120.00
C1—N1—H1N	117.00	C5—C4—H4	120.00
C2—N1—H1N	117.00	C3—C4—H4	120.00
C8—N2—H2N	115.00	C4—C5—H5	120.00
C1—N2—H2N	115.00	C6—C5—H5	120.00
C14—N3—H3N	118.00	C7—C6—H6	120.00
C15—N3—H3N	118.00	C5—C6—H6	120.00
C21—N4—H4N	118.00	C2—C7—H7	120.00
C14—N4—H4N	118.00	C6—C7—H7	120.00
S1—C1—N2	119.5 (2)	C10—C9—H9	120.00
N1—C1—N2	118.9 (2)	C8—C9—H9	120.00
S1—C1—N1	121.50 (19)	C11—C10—H10	120.00
C3—C2—C7	120.4 (3)	C9—C10—H10	120.00
N1—C2—C3	119.0 (3)	C10—C11—H11	120.00
N1—C2—C7	120.6 (3)	C12—C11—H11	120.00
C2—C3—C4	119.4 (4)	C11—C12—H12	120.00
C3—C4—C5	120.6 (4)	C13—C12—H12	120.00
C4—C5—C6	119.5 (4)	C12—C13—H13	120.00
C5—C6—C7	120.8 (4)	C8—C13—H13	120.00
C2—C7—C6	119.3 (4)	C15—C16—H16	120.00
N2—C8—C9	117.3 (2)	C17—C16—H16	120.00
C9—C8—C13	119.9 (3)	C18—C17—H17	120.00
N2—C8—C13	122.7 (2)	C16—C17—H17	120.00
C8—C9—C10	119.3 (3)	C17—C18—H18	120.00
C9—C10—C11	120.7 (3)	C19—C18—H18	120.00
C10—C11—C12	119.8 (3)	C20—C19—H19	120.00
C11—C12—C13	120.4 (3)	C18—C19—H19	120.00
C8—C13—C12	119.8 (3)	C15—C20—H20	120.00
S2—C14—N3	120.56 (19)	C19—C20—H20	120.00
S2—C14—N4	120.7 (2)	C23—C22—H22	120.00
N3—C14—N4	118.8 (2)	C21—C22—H22	120.00
N3—C15—C16	120.2 (3)	C22—C23—H23	120.00
C16—C15—C20	120.3 (3)	C24—C23—H23	120.00
N3—C15—C20	119.5 (3)	C25—C24—H24	120.00
C15—C16—C17	119.7 (4)	C23—C24—H24	120.00
C16—C17—C18	119.6 (4)	C24—C25—H25	120.00
C17—C18—C19	120.6 (3)	C26—C25—H25	120.00
C18—C19—C20	120.5 (4)	C21—C26—H26	121.00
C15—C20—C19	119.3 (3)	C25—C26—H26	121.00
			
Br1—Cu1—S1—C1	−7.29 (13)	C2—C3—C4—C5	−0.5 (6)
S2—Cu1—S1—C1	172.19 (12)	C3—C4—C5—C6	2.0 (7)
Br1—Cu1—S2—C14	−17.05 (13)	C4—C5—C6—C7	−1.1 (7)
S1—Cu1—S2—C14	163.48 (12)	C5—C6—C7—C2	−1.4 (6)
Cu1—S1—C1—N1	166.2 (2)	N2—C8—C9—C10	−177.1 (4)
Cu1—S1—C1—N2	−11.3 (3)	C13—C8—C9—C10	−1.0 (6)
Cu1—S2—C14—N3	176.2 (2)	N2—C8—C13—C12	177.9 (3)
Cu1—S2—C14—N4	−4.0 (3)	C9—C8—C13—C12	2.0 (6)
C2—N1—C1—S1	7.0 (4)	C8—C9—C10—C11	−0.4 (7)
C2—N1—C1—N2	−175.5 (3)	C9—C10—C11—C12	0.7 (7)
C1—N1—C2—C3	−130.1 (3)	C10—C11—C12—C13	0.3 (7)
C1—N1—C2—C7	53.2 (4)	C11—C12—C13—C8	−1.6 (6)
C8—N2—C1—S1	−169.9 (3)	N3—C15—C16—C17	−178.2 (3)
C8—N2—C1—N1	12.6 (5)	C20—C15—C16—C17	2.2 (5)
C1—N2—C8—C9	−150.7 (4)	N3—C15—C20—C19	179.5 (3)
C1—N2—C8—C13	33.2 (5)	C16—C15—C20—C19	−0.9 (5)
C15—N3—C14—S2	−1.0 (4)	C15—C16—C17—C18	−2.1 (5)
C15—N3—C14—N4	179.1 (3)	C16—C17—C18—C19	0.6 (6)
C14—N3—C15—C16	66.0 (4)	C17—C18—C19—C20	0.8 (6)
C14—N3—C15—C20	−114.4 (4)	C18—C19—C20—C15	−0.7 (5)
C21—N4—C14—S2	−172.0 (2)	N4—C21—C22—C23	179.8 (3)
C21—N4—C14—N3	7.9 (5)	C26—C21—C22—C23	−1.0 (5)
C14—N4—C21—C22	84.2 (4)	N4—C21—C26—C25	−178.9 (3)
C14—N4—C21—C26	−95.1 (4)	C22—C21—C26—C25	1.8 (5)
N1—C2—C3—C4	−178.8 (3)	C21—C22—C23—C24	−1.2 (6)
C7—C2—C3—C4	−2.0 (5)	C22—C23—C24—C25	2.5 (7)
N1—C2—C7—C6	179.6 (3)	C23—C24—C25—C26	−1.6 (7)
C3—C2—C7—C6	3.0 (5)	C24—C25—C26—C21	−0.5 (6)

### Hydrogen-bond geometry (Å, °)

**Table:**

*D*—H···*A*	*D*—H	H···*A*	*D*···*A*	*D*—H···*A*
N1—H1N···O1^i^	0.86	2.35	3.046 (4)	139
O1—H1O···S1^ii^	0.83	2.66	3.462 (3)	163
N2—H2N···Br1	0.86	2.59	3.435 (2)	169
N3—H3N···O1^iii^	0.86	2.16	2.957 (3)	155
N4—H4N···Br1	0.86	2.72	3.573 (2)	170
C13—H13···N1	0.93	2.58	3.000 (4)	108
C13—H13···S2^iv^	0.93	2.86	3.523 (3)	129
O1—H2O···CgD^iii^	0.80	2.78	3.306 (3)	125

Symmetry codes: (i) −*x*, −*y*+1, −*z*+1; (ii) *x*, *y*+1, *z*; (iii) −*x*+1, −*y*+1, −*z*; (iv) −*x*+1, −*y*, −*z*+1.
